# Supplementary material for: Impact of different landing heights on the contact force in the medial tibiofemoral compartment and the surrounding muscle force characteristics in drop jumps
Source: PLoS One. 2024 Jul 19;19(7):e0307538. doi: 10.1371/journal.pone.0307538 (PMC11259252; doi:10.1371/journal.pone.0307538)
Supplement: Attachment — Submitted filename: PLUS ONE. 5.5.2024 (ordibehesht 03).docx [file pone.0307538.s002.docx]

This article investigates Impact of different landing heights on the contact force in the medial tibiofemoral compartment and the surrounding muscle force characteristics in drop jumps.

The statistical analysis has been performed appropriately and the manuscript written in standard English.

 The comments below may help you rewrite the article.

**Abstract:**

- The conclusion is not clearly stated. In my opinion, it is better to state the conclusion of the study instead of practical suggestions for the study.

**Main text**

**Introduction**:

The problem statement should be revised. I did not understand what is the main difference between your study and previous studies.

- Why did you insert 16 participants? How was the sample size calculated?

***Subject Selection:***

"We recruited 16 male participants with 3-5 years of sports training experience……." What types of athletes did you include? Does the difference in the type of exercise interfere with the results of the study?line 123: insert refence of by Delp et al.

**Results:**

- lines 245-248: Specifically, the vGRF at a height of 0.15 m was significantly lower than that at other heights……… Additionally, the vGR increased significantly with landing height.

These two sentences have a repeated meaning. I think you can delete one.

***Discussion***:

The discussion section is too long. I think it is better to rewrite briefly and comprehensively.
