# Supplementary material for: Impact of different landing heights on the contact force in the medial tibiofemoral compartment and the surrounding muscle force characteristics in drop jumps
Source: PLoS One. 2024 Jul 19;19(7):e0307538. doi: 10.1371/journal.pone.0307538 (PMC11259252; doi:10.1371/journal.pone.0307538)
Supplement: Attachment — Submitted filename: Referee comments1.docx [file pone.0307538.s003.docx]

Dear Editor,

Thanks for providing this opportunity for me to review this manuscript.

The paper entitled "Impact of different landing heights on the contact force in the medial tibiofemoral compartment and the surrounding muscle force characteristics in drop jumps" is referring to an important factor in athletes which may be attributed to knee injuries and should be considered in prevention and treatment regimes.

**Abstract**

Please replace the word strengthen with improve ‘strengthen muscle coordination’ in the last line.

**Introduction**

- Line 85 please correct “leading a decrease in knee stiffness’

**Methods**

- Why did you recruit only male subjects, as many knee disorders such as PFA or PFPS is more common in female athletes?
- Were all the athletes engaging in one specific sport stream or different sports, please clarify, as different sport athletes will show different biomechanics of knee joint.
- using same shoes for athletes is controversial as shoe is one of key factors affecting GRF, muscle force and activations in drop jumps, unless you have provided a situation in which they have been using it at least 10 days before study.
- Please elaborate the position of cameras and which feature of tracker software did you use. (2D,3D,6Fd)
- Line 162 please correct” the captured motion capture data”

**Results**

Results is completely well-written

**Discussion**

- **Line 328 please use more proper statement**
- **Line 334 please correct** joint moment
- **Please elaborate if the force absorption was even between both legs or not**
- **Line 355 what do you mean by “consistent** with the findings of the present study”?
- **Line 365 better statement please**
- **Please rewrite the conclusion with consideration of what really your manuscript adds to previous studies with highlighting the impact in knee injuries**
- **The manuscript is very well-written and practical, wish you good luck**
