# Supplementary material for: Impact of different landing heights on the contact force in the medial tibiofemoral compartment and the surrounding muscle force characteristics in drop jumps
Source: PLoS One. 2024 Jul 19;19(7):e0307538. doi: 10.1371/journal.pone.0307538 (PMC11259252; doi:10.1371/journal.pone.0307538)
Supplement: Attachment — Submitted filename: Response to Reviewers.docx [file pone.0307538.s004.docx]

**Manuscript ID:** PONE-D-24-13422

**Title：**Impact of different landing heights on the contact force in the medial tibiofemoral compartment and the surrounding muscle force characteristics in drop jumps

***Response to the comments of Editor***

Dear Editor,

On behalf of my co-authors, we would like to express our sincere gratitude to you for giving us the opportunity to revise our paper. We are very appreciative of the constructive comments and suggestions provided by the editors and reviewers for our paper "Impact of different landing heights on the contact force in the medial tibiofemoral compartment and the surrounding muscle force characteristics in drop jumps" (ID: PONE-D-24-13422). We carefully reviewed the feedback from the reviewers and yourself, and made revisions, which are marked in yellow in the paper. The revised version is attached, and we would like to submit it for your consideration.

We would like to briefly answer your questions and comments:

(1) Regarding the format of the manuscript: The manuscript has been meticulously modified in accordance with the style requirements of PLOS ONE. If there are still any inconsistencies, we would appreciate it if you could point them out, and we will certainly take the suggestions seriously and make the necessary revisions.

(2) With regard to the copyright issue of Figure 2: The authors have chosen to remove Figure 2. Figure 2 is a screenshot from the OpenSim software, and copyright permission cannot be obtained at present. In addition, the description of Figure 2 in the paper is clear enough, and appropriate references have been made, so we are considering removing Figure 2 from the article and submission. Consequently, we have opted to remove Figure 2 from the manuscript . The updated manuscript, which now excludes Figure 2, has been resubmitted, and the figure caption has been revised to ensure coherence and integrity. Specific details on this modification can be found in the latest uploaded "manuscript" file.

(3) Regarding the verification of the reference list: The authors have checked and ensured the completeness and accuracy of your reference list. This paper does not reference any retracted articles.

Thank you again for your patience and assistance. We look forward to your further guidance to ensure that our manuscript meets the requirements of the journal. We are eagerly awaiting your response

Yours sincerely,

Lijun Hua

Department：College of Physical Education and Training

Harbin Sport University,

Harbin 150008, Heilongjiang, China

Email: hualijun@hrbipe.edu.cn

***Response to the comments of Reviewers***

***Response to the comments*** ***of Reviewer 1***

Dear Reviewer,

We deeply appreciate your meticulous review of our paper. Following your suggestions, we have revised the relevant sections and made certain changes to the manuscript. These alterations do not affect the core content or overall structure of the paper. We are profoundly grateful for your professionalism and dedication, and hope that the revised paper gains your approval. All changes are detailed in the following **"Response"** section and the revised content is highlighted in **yellow** in the original manuscript for easy reference. We hope our revised manuscript will be acceptable to you.

We look forward to your valuable feedback and suggestions on our revised manuscript. We sincerely appreciate the time and effort invested by the reviewer in evaluating our paper and look forward to receiving your additional feedback or suggestions.

Sincerely,

Lijun Hua

In the subsequent section, a thorough response is offered in relation to the comments you have provided.

**Comments:**

**(1)*Abstract***

***The conclusion is not clearly stated. In my opinion, it is better to state the conclusion of the study instead of practical suggestions for the study*”**

**Response:**

Thank you for providing valuable feedback. We have incorporated the revisions into the conclusion of the "Abstract" section of the manuscript. The specific modifications are as follows:

1) Stating research findings: During drop jumps, both the knee moment and the MCF exhibited coordinated changes.

2) Removal of practical suggestions: We have deleted the provided practical operational suggestions and focused on the research conclusions themselves.

3) Highlighting the significance of the study: Emphasis has been placed on the impact of different drop jump heights on the muscles around the knee joint, as well as the specific muscular responses and their importance for injury prevention.

The revised conclusion in the abstract is as follows:

"Thus, both the knee moment and the MCF exhibited similar coordinated changes during drop jumps, indicating that these may be adaptive movement strategy. The impact of varying drop jump heights on muscle groups around the knee joint varied suggest that different heights induce specific muscular responses and improve muscle coordination to prevent knee joint injuries."

**#**Please note that the specific revisions mentioned can be found in the "Manuscript" file, lines 28-32.

**#**Alternatively, you can refer to the "Revised Manuscript with Track Changes" file , where the indicated lines 28-33 are highlighted with a yellow background by the author.

**Comments**

**(2):*Main text Introduction***

***The problem statement should be revised. I did not understand what is the main difference between your study and previous studies.***

**Response:**

We appreciate your valuable feedback and in-depth insights. Your suggestion to highlight the differences between our research and previous studies is highly constructive. We agree to make the revisions and have incorporated them into the Introduction section.

Specific modifications are as follows:

1) Emphasizing the differences between our research and previous studies: It is explicitly pointed out that there is a scarcity of research on the tibiofemoral joint's medial compartment force (MCF) during jump landing movements. Previous studies have relied predominantly on the knee adduction moment (KAM).

The specific revisions are as follows:

"Despite its importance, research specifically focusing on the tibiofemoral joint's medial compartment force (MCF) during DJ movements is scarce. Previous studies have often relied on the KAM as a proxy, which does not fully capture the load distribution characteristics of the medial compartment."

**#**Please refer to the specific modifications in the "Manuscript" file, lines 64-68. **#**Alternatively, you can find the exact changes in the "Revised Manuscript with Track Changes" file, in the "No Markup" mode, where the highlighted sections can be found in lines 67-70 with a yellow background.

2) Strengthening the research objectives: This study aims to provide a more precise understanding of knee joint loading by analyzing the impact of different landing heights on MCF, KAM, and muscle characteristics. The goal is to offer guidance for athletes in selecting appropriate jump landing heights.

The specific revisions are as follows:

"By analyzing the impact of different landing heights on these characteristics ,this study aims to provide a more precise understanding of knee joint loading and the role of muscle forces during DJs. This will offer valuable insights for athletes in selecting suitable DJ landing heights to minimize knee joint load, enhancing current knowledge on DJ training and its implications for knee joint health. "

**#**Please refer to the specific modifications in the "Manuscript" file, lines 93-97. **#**Alternatively, you can refer to the "Revised Manuscript with Track Changes" file , where the indicated lines 100-106 are highlighted with a yellow background by the author.

**Comments:**

**(3)*Subject Selection: Why did you insert 16 participants? How was the sample size calculated?***

**Response:**

Thank you for providing thoughtful and constructive feedback. Regarding the calculation of sample size, we used GPower to estimate the sample size and consulted relevant literature (as listed below). The number of participants ranged from approximately 10 to 16. However, we did not provide detailed explanations in the manuscript. We appreciate your attentive reminder and suggestion. We have decided to accept the revision and will add the method for calculating the sample size in the article.

The specific revisions are as follows:

"A prediction for the sample size was carried out using GPower 3.1.9.7, with a significance level (α) of 0.05 and a statistical power of 80%. The analysis determined that a minimum sample size required was 13 individuals. "

**#**Please refer to the specific modifications in the "Manuscript" file, lines 106-108. **#**Alternatively, you can refer to the "Revised Manuscript with Track Changes" file , where the indicated lines 117-119 are highlighted with a yellow background by the author.

Below are the references:

Peng, H. T. (2011). Changes in biomechanical properties during drop jumps of incremental height. The Journal of Strength & Conditioning Research, 25(9), 2510-2518.(This article also selected 16 young, healthy college students.)

Sinclair J, Hobbs SJ, Selfe J. The Influence of Minimalist Footwear on Knee and Ankle Load during Depth Jumping. Research in Sports Medicine. 2015;23(3):289-301. doi: 10.1080/15438627.2015.1040917.(This article selected 10 male participants.)

Tsatalas, T., Karampina, E., Mina, M. A., Patikas, D. A., Laschou, V. C., Pappas, A., ... & Giakas, G. (2021). Altered drop jump landing biomechanics following eccentric exercise-induced muscle damage. Sports, 9(2), 24.(This article selected 15 regional level athletes.)

**Comments:**

**(4)*"We recruited 16 male participants with 3-5 years of sports training experience……." What types of athletes did you include? Does the difference in the type of exercise interfere with the results of the study?***

**Response:**

We apologize for overlooking the participants' types of sports and whether it influenced the research results. We appreciate the reviewer's suggestion and have made revisions accordingly. Our participants primarily come from sports such as basketball, volleyball, and soccer, which involve frequent jumping and landing movements similar to those in our study. This ensures the representativeness and consistency of the research findings. Furthermore, we have added relevant content to the manuscript.

The specific revisions are as follows:

"The participants predominantly engaged in sports such as basketball, volleyball, and soccer, which necessitate frequent jumping and landing movements analogous to the drop jumps executed in this study. "

**#**Please refer to the specific modifications in the "Manuscript" file, lines 109-111. **#**Alternatively, you can refer to the "Revised Manuscript with Track Changes" file , where the indicated lines 120-122 are highlighted with a yellow background by the author.

**Comments:**

**(5)*l******ine 123: insert refence of by Delp et al.***

**Response:**

Thank you for your careful review. We apologize for the oversight in missing references in the article. We sincerely apologize for the mistake. We have taken your suggestion into account and have added the reference by Delp et al.

The specific reference is as follows:

Delp SL, Anderson FC, Arnold AS, Loan P, Habib A, John CT, et al. OpenSim: open-source software to create and analyze dynamic simulations of movement. IEEE Trans Biomed Eng. 2007;54(11):1940-50. Epub 2007/11/21. doi: 10.1109/TBME.2007.901024. PubMed PMID: 18018689.

**#**Please refer to the specific modification at Line 130 in the "Manuscript" file. **#**Alternatively, you can find the exact modification location in the "Revised Manuscript with Track Changes" file at line 142. The author has highlighted it with a yellow background for better visibility.

**Comments:**

**(6)*Results:***

***lines 245-248: Specifically, the vGRF at a height of 0.15 m was significantly lower than that at other heights. Additionally, the vGRF increased significantly with landing height.These two sentences have a repeated meaning. I think you can delete one.***

**Response**:

Thank you for your careful review. After consideration, we have decided to remove the duplicate sentence in order to avoid repetition. Thank you again for your diligent and thorough review of the manuscript.

#For the specific modification location, please refer to Line 248 in the "Manuscript" file, where it has already been deleted.

#Alternatively, you can refer to the "Revised Manuscript with Track Changes" file , where the indicated lines 263-264 .

**Comments:**

**(7)*Discussion:***

***The discussion section is too long. I think it is better to rewrite briefly and comprehensively.***

**Response**:

Thank you for your valuable suggestions. We have realized that the discussion section is somewhat bulky, so based on your feedback, we have made modifications and deletions to some sentences in order to refine the discussion while maintaining the overall structure. We will further streamline the discussion to ensure that all content is closely tied to the main theme and is concise. Once again, we appreciate your patient review and valuable suggestions.

Regarding your feedback, let me briefly explain the modifications made to the "Discussion" section in the manuscript.

Firstly, we have eliminated parts of the discussion section that contain semantic repetition. This mainly includes:

Reduction of the simple understanding and comparison of the data in Table 1 in the manuscript. - Partial removal of excessive descriptions of the body's center of gravity in the text.

Deletion of repetitive conclusions and viewpoints in the manuscript.

Reduction of redundant statements about the limitations of the study in the manuscript. As well as some other deletions in specific parts.

**#**Please refer to the "Revised Manuscript with Track Changes" file for details of the deleted content in the discussion section.

Secondly, in the discussion section, each sentence has been refined and rewritten to make it more concise and fluent. For specific modification content and location, **#**Please refer to the "Revised Manuscript with Track Changes" file for details of the discussion section . The author has highlighted the modifications with a yellow background.

***Response to the comments of Reviewer 2***

Dear Reviewer,

We would like to extend our sincere gratitude for your thorough evaluation of our manuscript. In response to your insightful recommendations, we have carefully revised the pertinent sections while ensuring that the core content and overall structure of the paper remain unchanged. We greatly value your expertise and commitment, and we are hopeful that the revised paper meets with your approval. Detailed information regarding all modifications is provided in the "**Response**" section, and the revised content is highlighted in **yellow** within the original manuscript for your convenience. We are optimistic that our revised manuscript aligns with your expectations.

Your expertise and dedication to the review process are deeply appreciated, and we sincerely hope that the revised manuscript is in line with your expectations.

Sincerely,

Lijun Hua

In the subsequent section, a thorough response is offered in relation to the comments you have provided.

**Comments**:

**(1)*Abstract***

***Please replace the word strengthen with improve ‘strengthen muscle coordination’ in the last line.***

**Response**:

Thank you for the valuable suggestion. We accept this revision and will replace "strengthen" with "improve" .

"The impact of varying drop jump heights on muscle groups around the knee joint varied suggests that different heights induce specific muscular responses and improve muscle coordination to prevent knee joint injuries."

**#**The specific modifications are located in "Manuscript" file at Line30-32.

**#**Alternatively, you can refer to the "Revised Manuscript with Track Changes" file , where the indicated lines 30-33 are highlighted with a yellow background by the author.

**Comments**:

**(2)*Introduction***

***Line 85 please correct "leading a decrease in knee stiffness’***

***At higher drop jump heights, the increased impact forces can lead to changes in muscle activation patterns, resulting in a decrease in knee stiffness.***

**Response**:

Thank you for the valuable comments from the reviewer. Based on your suggestions, we have made revisions.

The specific changes are as follows:

"At higher drop jump heights, the increased impact forces can lead to changes in muscle activation patterns, resulting in a decrease in knee stiffness."

**#**For the specific changes, please refer to Lines 84-85 in the "Manuscript" file. **#**Alternatively, you can refer to the "Revised Manuscript with Track Changes" file , where the indicated lines 91-93 are highlighted with a yellow background by the author.

**Comments**:

**(3)*Methods***

***Why did you recruit only male subjects, as many knee disorders such as PFA or PFPS is more common in female athletes?***

**Response**:

Dear reviewer, thank you for your valuable suggestion. Regarding your concerns, we completely understand the rationale behind your question. The reason for recruiting only male subjects was to control for the variability in biomechanical responses related to gender, ensuring the accuracy of our research findings. We are aware that many knee disorders, such as patellofemoral pain syndrome (PFPS) and patellofemoral arthritis (PFA), are more common in female athletes. In our future studies, we will definitely include female subjects to addressing this limitation and gaining a comprehensive understanding of the biomechanics of the knee joint across different genders. This aspect has also been addressed in line 428 of the manuscript. Once again, thank you for your guidance and attention. We will strive to further improve our research.

**Comments**:

**(4)*Were all the athletes engaging in one specific sport stream or different sports, please clarify, as different sport athletes will show different biomechanics of knee joint.***

**Response**:

Dear reviewer, thank you very much for your query and attention. Regarding your question, we are pleased to clarify that the athletes recruited for this study were from various sports including soccer, basketball, and track and field, among others. This diversity in selection aimed to capture a range of biomechanical responses associated with different sports. In response to your suggestion, we have included the statement "The participants predominantly engaged in sports such as basketball, volleyball, and soccer, which necessitate frequent jumping and landing movements analogous to the drop jumps executed in this study" in the manuscript to adequately address your query. Once again, thank you for your guidance, and we will strive to further enhance the quality of our research.

#The specific modification has been made in lines 109 to 111 of the manuscript.

#Alternatively, you can refer to the "Revised Manuscript with Track Changes" file , where the indicated lines 120-122 are highlighted with a yellow background by the author.

**Comments**:

**(5)*using same shoes for athletes is controversial as shoe is one of key factors affecting GRF, muscle force and activations in drop jumps, unless you have provided a situation in which they have been using it at least 10 days before study.***

**Response**:

Dear reviewer, we would like to sincerely thank you for your valuable suggestion. In response to your concern, we would like to clarify that during the drop jump testing, all athletes were required to wear the same type of shoes to standardize external conditions and minimize the potential impact of footwear on ground reaction forces (GRF), muscle force, and activation. It is true that we did not mandate all participants to have worn the provided shoes for at least 10 days prior to the study, which would have mitigated any initial discomfort or adaptation effects associated with new shoes. We believe that this is a very valuable suggestion, and we will consider implementing this requirement in future studies. Once again, thank you for your insightful input, and we will continue to strive for rigor and reliability in our research.

**Comments**:

**(6)*Please elaborate the position of cameras and which feature of tracker software did you use. (2D,3D,6Fd)***

**Response**:

Dear reviewer, thank you very much for your attention. We have placed eight cameras in our laboratory, positioned at a higher horizontal level with respect to the rectangular ceiling of the laboratory. Each edge is equipped with two cameras. This setup is designed to capture the 3D motion trajectory of the participants in the experimental space. In the data analysis, we used the Qualisys Track Manager software (specifically mentioned in the manuscript at line128) to determine the motion trajectory and features of the participants. We hope that this explanation can better address your question. Corresponding modifications have also been made in the manuscript.Once again, thank you for your attention and suggestion.

The specific changes are as follows:

"The experiment employed eight 600 series high-speed cameras (200 Hz, Qualisys, Sweden), with two cameras mounted on each of the laboratory's four walls at an equal elevation."

**#**The specific modifications are located in "Manuscript" file at Line121-123.

**#**Alternatively, you can refer to the "Revised Manuscript with Track Changes" file , where the indicated lines 132-134 are highlighted with a yellow background by the author.

**Comments**:

**(7)*Line 162 please correct” the captured motion*** ***capture data”***

**Response**:

Dear reviewer, thank you for pointing that out. The term "the captured motion capture data" in line 162 contains redundancy, and we will make the necessary correction. We appreciate your careful review and valuable suggestion.

The specific changes are as follows:

"First, the captured motion data were converted and processed by applying low-pass filters of 10 Hz and 100 Hz to the kinematic and kinetic parameters, respectively."

**#**The specific modifications can be found in the "Manuscript" file at Line 165-166. **#**Alternatively, you can refer to the "Revised Manuscript with Track Changes" file , where the indicated lines 180-182 are highlighted with a yellow background by the author.

**Comments:**

**(8)*Results***

***Results is completely well written***

**Response**:

we appreciate the positive feedback from the reviewer regarding the clarity and accuracy of our results section. We are pleased with the clear and accurate presentation of our results.

**Comments:**

**(9)*Discussion***

***Line 328*** ***please use more proper statement***

**Response**:

Thank you for your careful review and feedback. We have made the following changes: we replaced "demonstrated" with "indicated" to convey a more formal and academic tone. We also acknowledged the non-standard use of "inversion-eversion" and replaced it with "KAM," which stands for knee adduction moment, for a more standardized expression.

The specific changes are as follows:

"Previous studies have indicated that higher drop landing heights significantly increase the peak KFM and KAM during DJs"

**#**The specific modifications can be found in "Manuscript" file at Line 322-323. #Alternatively, you can refer to the "Revised Manuscript with Track Changes" file , where the indicated lines 340-342 are highlighted with a yellow background by the author.

**Comments**:

**(10)*Line 334 please correct joint moment，******Please elaborate if the force absorption was even between both legs or not***

**Response**:

Thank you very much for taking the time to review our manuscript and provide valuable feedback.In response to your suggestion, we have made modifications to "joint moment," changing it to "joint moments" to ensure the use of the plural form, as the knee joint involves multiple directions of moments. However, we are concerned that we may not have fully understood your intended meaning. If this modification does not meet your expectations, please do not hesitate to guide us, and we will make the necessary adjustments promptly. The rest of the passage remains unchanged to ensure the smoothness and accuracy of expression.

Regarding the issue you raised in your comments about the evenness of force absorption between both legs, in this study, we primarily focus on the single-leg landing and takeoff during the deep jump movement, rather than the force absorption between both legs. Perhaps we did not specifically emphasize this characteristic in the original manuscript, resulting in this oversight. In response to your feedback, we have revised the manuscript to include a detailed description of the single-leg landing and takeoff movement.

The specific changes are as follows:

" In single-leg drop Jumps, as the primary site for absorbing and dispersing forces, the knee joint must increase joint moments to cope with this increased impact force. The measured vGRFs confirmed this trend, with a significantly increase observed at higher DJ landing heights, resulting in a greater impact force on the body."

**#**The specific modifications can be found in "Manuscript" file at Line 328-332. **#**Alternatively, you can refer to the "Revised Manuscript with Track Changes" file , where the indicated lines 347-352 are highlighted with a yellow background by the author.

**Comments**:

**(11)*Line 355 what do you mean by "consistent with the findings of the present study”?***

**Response**:

Dear Reviewer,Thank you very much for your valuable suggestions on our paper. Regarding your proposed modification, we have revised "consistent with the findings of the present study" to "which aligns with the observations of this study" as per your advice. This modification has made our expression clearer and more coherent, enhancing the logical flow and highlighting the consistency between the relevant research results and the observations of our study.

The specific changes are as follows:

"Related studies on walking, running, and lateral movements have also indicated larger medial compartment contact forces in the MCFfy , which aligns with the observations of this study."

**#**The specific modifications can be found in "Manuscript" file at Line 345-347. **#**Alternatively, you can find the specific modifications in the "Revised Manuscript with Track Changes" file, in the "No Markup" mode, on lines 372-375, where the authors have highlighted the changes using a yellow background.

**Comments**:

**(12)*Line 365 better statement please***

**Response**:

Dear Reviewer,Thank you very much for reviewing our manuscript and providing your feedback. In response to your suggestion, we have made the following adjustments: we have modified "may also be a factor" to "may also play a role" to make the language more formal and clear; we have changed "is a phenomenon in which" to "refers to" to simplify the expression and improve readability; we have added "This mechanism" to refer to the previous phenomenon, enhancing the coherence of the sentence; and we have replaced "reducing harm" with "thereby reducing the potential for injury" to make the statement more specific and clear.

The specific changes are as follows:

"Additionally, the phenomenon of muscle pre-activation may also play a role, which is observed in movements such as jumping and walking. Muscle pre-activation refers to the central nervous system activates muscles in advance to prepare for the post-landing movement, This mechanism can trigger protective muscle strategies in unforeseen circumstances, thereby reducing the potential for injury ."

**#**The specific modifications can be found in "Manuscript" file at Line 353-358. **#**Alternatively, you can refer to the "Revised Manuscript with Track Changes" file , where the indicated lines 381-388 are highlighted with a yellow background by the author.

**Comments**:

**(13)*Please rewrite the conclusion with consideration of what really your manuscript adds to previous studies with highlighting the impact in knee injuries***

**Response**:

Dear Reviewer,Thank you very much for your valuable suggestions on our paper. In response to your feedback, we have rewritten the conclusion. We highlighted the unique contribution of this study and emphasized its impact on knee injuries. This adjustment has strengthened the logic and clarity of the conclusion, making it more comprehensive and persuasive. Additionally, we specifically connected the relevant research findings to practical applications, such as sports training and rehabilitation programs, to emphasize their significance and practicality.

The specific changes are as follows:

"In this study, we found that increased landing heights significantly augmented medial compartment forces (MCF) and influenced knee joint moments (KFM, KAM, and KRM) in patterns similar to the changes in MCF components (MCFfx, MCFfy, and MCFfz) throughout the entire DJ process. This suggests that the knee joint segments effectively coordinate to maintain stability and movement efficiency during complex actions like DJs, representing an adaptive movement strategy. Furthermore, the impact of different landing heights on major muscle groups around the knee, such as the quadriceps, was evident. Higher jump heights notably increased the strength of the VI and VL, while the strength of the VM decreased. These findings highlight the need for a gradual increase in jump height combined with reinforced muscle pre-activation and balance coordination training to mitigate the risk of knee injuries. This study contributes to the existing body of knowledge by emphasizing the importance of controlled jump training to enhance knee joint stability and prevent injuries, which has significant implications for athletic training and rehabilitation programs."

**#**The specific modifications can be found in "Manuscript" file at Line 439-452.

**#**Alternatively, you can refer to the "Revised Manuscript with Track Changes" file , where the indicated lines 501-514 are highlighted with a yellow background by the author.

**Comments**:

**(14)*****The manuscript is very well written and practical, wish you good luck***

**Response**:Thank you very much for the positive feedback from the reviewer. We will continue to strive for excellence and we appreciate your well wishes.We deeply appreciate the time and effort dedicated by the reviewer to assess our manuscript. We eagerly anticipate any further feedback or suggestions.
